# Development of a new quantitative gas permeability method for dental implant-abutment connection tightness assessment

**DOI:** 10.1186/1475-925X-10-28

**Published:** 2011-04-14

**Authors:** Jacques-Henri Torres, Michael Mechali, Olivier Romieu, Paul Tramini, Sylvie Callas, Frédéric JG Cuisinier, Bernard Levallois

**Affiliations:** 1 EA42503, LBN, Université Montpellier 1, Montpellier, France; 2 Matériaux Hybrides et Nanocomposites, GES - UMR CNRS 5650, Université Montpellier 2, Montpellier 34000, France

## Abstract

**Background:**

Most dental implant systems are presently made of two pieces: the implant itself and the abutment. The connection tightness between those two pieces is a key point to prevent bacterial proliferation, tissue inflammation and bone loss. The leak has been previously estimated by microbial, color tracer and endotoxin percolation.

**Methods:**

A new nitrogen flow technique was developed for implant-abutment connection leakage measurement, adapted from a recent, sensitive, reproducible and quantitative method used to assess endodontic sealing.

**Results:**

The results show very significant differences between various sealing and screwing conditions. The remaining flow was lower after key screwing compared to hand screwing (p = 0.03) and remained different from the negative test (p = 0.0004). The method reproducibility was very good, with a coefficient of variation of 1.29%.

**Conclusions:**

Therefore, the presented new gas flow method appears to be a simple and robust method to compare different implant systems. It allows successive measures without disconnecting the abutment from the implant and should in particular be used to assess the behavior of the connection before and after mechanical stress.

## Background

Dental implant systems presently found on the market are mainly made of two pieces: the implant itself (placed inside the alveolar bone) and the abutment (which goes through the gum and supports the prosthesis). The connection between those two pieces appears to be a key point for implant success. Essentially, besides mechanical considerations, a gap between those two pieces may allow bacterial proliferation, inflammation and peri-implant bone loss [1-6]. It appears an important challenge to assess the tightness of this connection. This has already been tested by:

- showing microbial leakage at the implant-abutment interface in patients [7,8] and *in vitro *[6,9-15];

- placing a color marker between the implant and the abutment and measuring its leakage by spectrophotometry [16,17];

- and, more recently, studying tightness against endotoxins [18].

These techniques hardly provide a quantitative and reproducible way to measure the leakage, as it was demonstrated in endodontics [19]. Indeed, many techniques have been used to investigate the sealing ability of root filling procedures and materials. Some leakage investigations, like dye spectrometry [20], fluid filtration [21], and electrochemistry [22], are considered to provide pure quantitative data. Other studies using bacteria are essentially qualitative [23,24]. However, the majority of leakage tests are related to linear measurement of tracers like coloring agents and radioisotopes [25], which give semi-quantitative data [26]. Despite the long experience of measuring tightness in this field, *in vitro *assessment of sealability has lost its credibility [27]. Among the leakage studies, tracer diffusion studies are the more frequent but have been demonstrated to have false conclusions [28] and results to be very driven by presence of air entrapped. These critics could be applied to implants leakage studies using tracers or bacteria entrapped in the inner part of the implant.

Recently, a gas flow test was shown to be a sensitive, reproducible and quantitative method to assess endodontic sealing [29]. The aim of the present engineering contribution was to adapt this gas permeability technique to implant-abutment connection leakage, in order to provide a new quantitative and reproducible tool for further investigations. Such a method has never been used in the field of implantology for assessing implant-abutment microgap before.

## Methods

**Figure 1:**
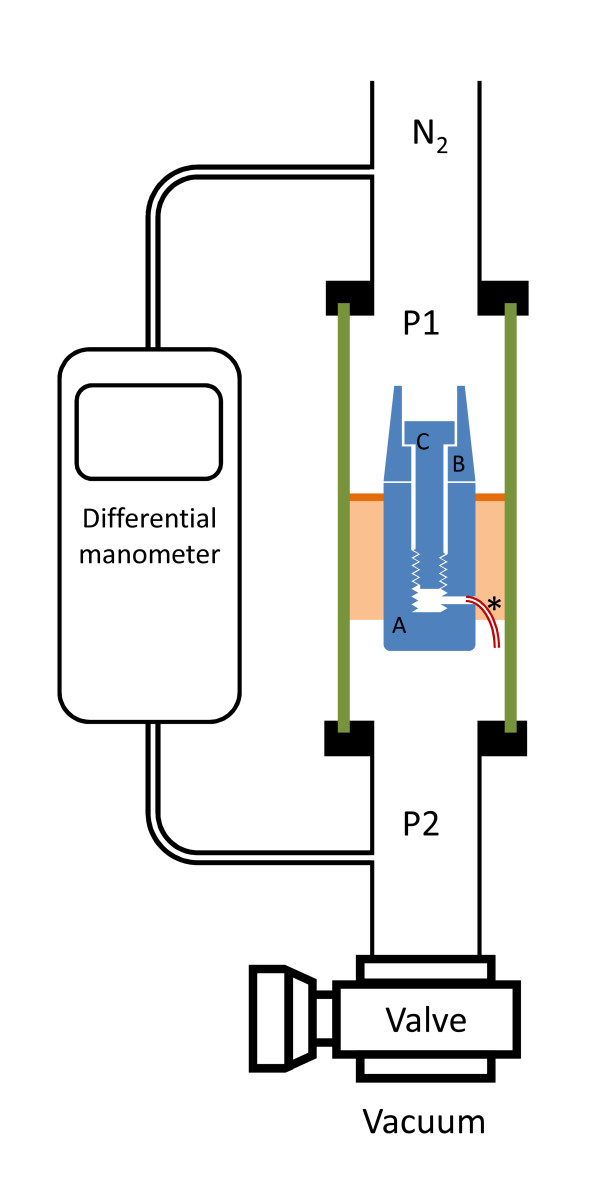
**Experimental set-up**. Nitrogen leaks from chamber P1 to chamber P2 through the gap between implant (A) and abutment (B) and between the screw (C) and the abutment, then through a plastic tube (*) connecting the internal chamber of the implant with chamber P2. In the glass tube (green), the implant is sealed in epoxy adhesive (pink) and the tightness is ensured with wax (brown).

The experimental set-up used is similar to the set-up used for assessing endodontic leakage [20]. The implants were positioned in an experimental chamber between atmospheric (P1) and negative (P2) nitrogen pressure. Gas flow was assessed measuring the pressure difference between P1 and P2 with a differential pressure gauge (Testo 526, Forbach, France). After vacuum was completed, the needle valve was closed (see Figure 1). Initial pressure difference was about 1010 hPa. Pressure difference versus time was recorded.

**Figure 2:**
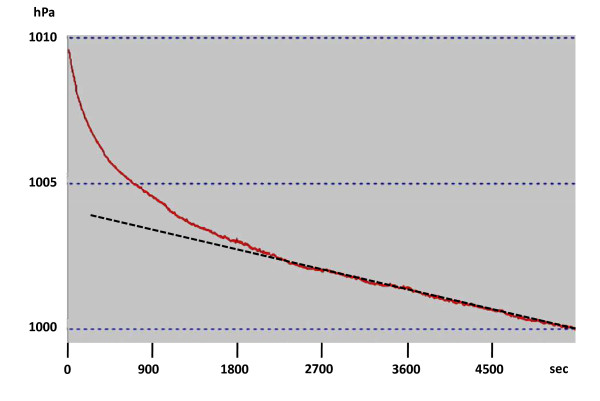
**Example of a recorded curve**. Progressive decrease in pressure: initial drop is followed by a straight line (black dotted), the slope of which was determined by the operator.

Typical pressure curves showed an initial drop followed by a second regimen appearing as a straight line (Figure 2). This latter regimen is due to a pressure difference low enough, compared to atmospheric pressure: the nitrogen flow is not related any longer to pressure difference, but only to the importance of leakage, and obeys Knudsen's law [30]. The slope of the line was measured by an operator, and recorded. In order to assess the reproducibility of the slope determination by the operator, 10 curves were read twice: another operator drew lots 10 curves from among all the recorded curves. The first operator had to perform again slope determination for these curves, blindly. Reproducibility of the measurements was tested by a Kendall's coefficient of concordance.

First of all, three types of negative assays were performed to appraise the gas tightness of the employed materials:

- a blind-ended glass test tube was used to assess the gas tightness of the experimental chamber;

- a double-ended opened glass tube sealed on one side with a plug of epoxy glue to assess the gas tightness between the glue and the glass;

- a double-ended opened glass tube sealed on one side with a plug of wax to assess the gas tightness of wax.

Under water spray, five One Morse implants (One System Implant, Cannes France), 4.3 mm diameter and 12 mm length, were drilled a hole to the apical part of the screw cavity. A plastic tube was inserted in the hole. The implants were partially embedded in epoxy glue (Araldite 2012, Huntsman Polyurethanes, Everberg, Belgium) and sealed in a glass tube, allowing free spaces around the abutment connection and around the tube. Wax (Purple wax, GC Europe, Leuven, Belgium) was used to seal implant to glue, and to block the various gaps to be assessed.

**Figure 3:**
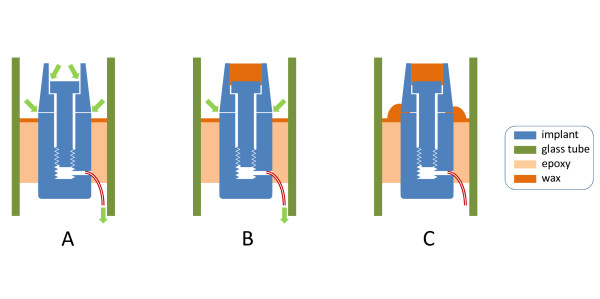
**Test conditions applied**. Test conditions applied for each implant (green arrows indicate the possible leakage paths): A) abutment manual screwing. Nitrogen flow may occur at both gaps: between screw and abutment and between abutment and implant collar (assay 1). B) screw hole blocked with wax; manual (assay 2) or key (assay 3) screwing. C) abutment key screwing, screw hole and implant to abutment connection blocked with wax (as a negative test).

Each implant was tested 4 times, respectively after (Figure 3):

Assay 1) abutment manual screwing (by one operator)

Assay 2) abutment manual screwing (by one operator), screw hole blocked with wax

Assay 3) abutment key screwing (35 Ncm), screw hole blocked with wax

Assay 4) abutment key screwing, screw hole and implant connection blocked with wax (as a negative test)

Assay number 3 was performed 10 times for one of the implants to assess the reproducibility of the method.

ANOVA of repeated measures was performed globally to test whether the measured slopes for the 4 assays were globally different. After a logarithmic transformation, data followed a normal repartition. Pairwise comparisons between each assay were tested taking into account Bonferroni correction at alpha = 0.05 (significance).

## Results

### Negative tests

**Figure 4:**
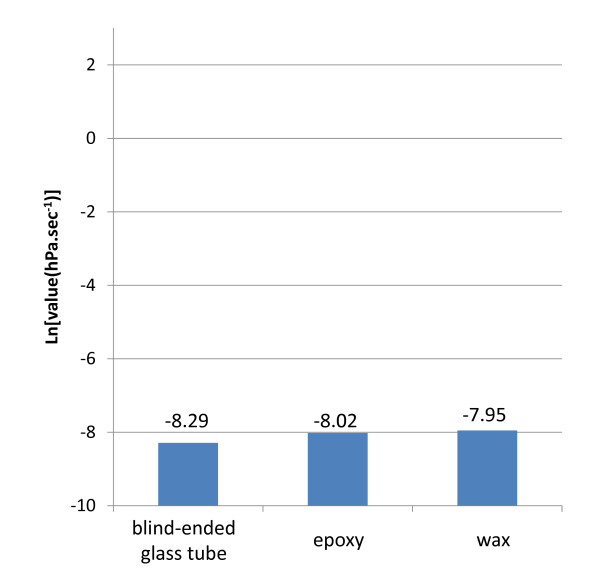
**Experimental set-up tightness**. Three tests were realized to assess experimental set-up tightness: -blind-ended glass tube; - epoxy: a double-ended opened glass tube sealed on one side with a plug of epoxy glue; - wax: a double-ended opened glass tube sealed on one side with a plug of wax.

The negative tests performed proved that the experimental setup and the various materials used to stop nitrogen leaking show convenient gas tightness (Figure 4). The slope of the blind-ended glass tube (0.000252 hPa.sec^-1^, or e^-8.29 ^hPa.sec^-1^) gave an idea of the maximal tightness of the experimental setup i.e. the remaining leakage when the system is totally closed. Measures of tightness obtained with the epoxy glue and the blind-ended glass tube were in the same range (e^-8.02 ^and e^-7.95 ^hPa.sec^-1 ^respectively).

### Reproducibility

The slope determination reproducibility for 10 curves, given by the Kendall's coefficient (W) of concordance test, was 0.9 (Kendall test), p = 0.0004. Reproducibility of the method has been calculated by repeating 10 times the measure on one implant and the abutment screwed with a dynamometric key at a torque value of 35 N.cm, and screw hole blocked with wax; the coefficient of variation was 1.29%, which is very low and is usually associated with reproducible methods of measurement.

### Dental implant-abutment assays

**Figure 5:**
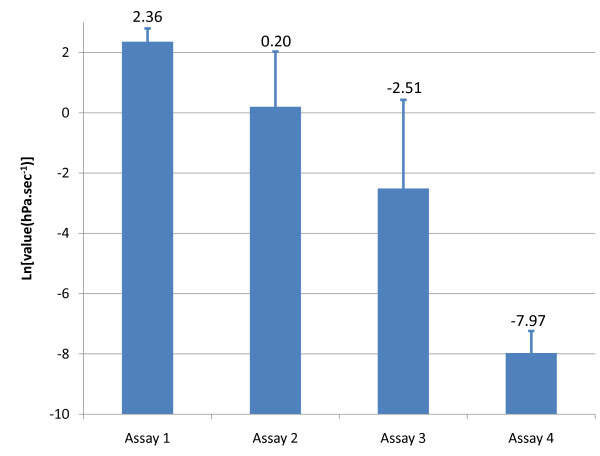
**Gas leaking in four conditions**. Gas leaking for the tested implants (values are indicated up bars): Assay 1, abutment manual screwing; Assay 2, abutment manual screwing, screw hole blocked with wax; Assay 3, abutment key screwing, screw hole blocked with wax; Assay 4, negative test with abutment key screwing, both screw hole and implant to abutment connection blocked with wax.

The results of the four assays (Figure 5) globally showed a very significant difference between the various sealing conditions. As could be expected, a lower flow was observed after having filled the screw hole with wax (though the difference was not significant between assay 1 and assay 2) (Table 1). Also, the dental implant-abutment remaining flow was lower after key screwing compared to hand screwing (p = 0.03). Finally, the implant-abutment flow after key screwing showed to remain different from the negative test (p = 0.0004). This observation suggests the persistence of leakage after key screwing in this sort of implant.

**Table 1:** Pairwise comparisons between each assay

Vs	Assay 4	Assay 3	Assay 2
	
Assay 1	p = 0.0001*	p = 0.02*	p = 0.06
Assay 2	p = 0.0001*	p = 0.03*	

Assay 3	p = 0.0004*		

## Discussion

Clinically, existence of a gap does not mean necessarily bone loss. In particular, it was shown that micro-movement may have a higher influence than leakage [31]. Furthermore, in this gas permeability model, the size of porosity cannot be assessed precisely. The smaller capillary measurable with gas permeability has an internal diameter of 10 μm (data not shown). This is much bigger than bacteria or even than endotoxin.

On the other hand, this assay only assesses the global tightness of the connection. The possible spaces around the implant collar which would not communicate with the inner part of the implant do not influence the result, though they can have a major clinical role. Despite this, leakage can be considered as a good marker of the machining quality.

Unlike in the color marker methods, this technique allows successive measures of the different interfaces (screw hole, collar) without re-opening the connection. This characteristic could be very useful for assessing different treatments of the implant connection such as screwing torque control, chemical stress or mechanical stress. Clinically, tightness could be improved by different ways apart from the quality of machining, for instance by the use of a sealent such as GapSeal^® ^(Hager Werken), though the authors did not find any scientific publication about this product using PubMed.

Using gas leakage allows a precise physical and reproducible value independent of water-wetting properties of the materials tested. The global leak measure obtained can be compared, for simplification and calibration, to an "equivalent capillary" with a length-diameter couple.

## Conclusions

This gas flow method appears to be a simple way to compare different implant systems. It seems to be better than the color marker techniques because it allows successive measures without disconnecting the abutment from the implant and is not subjected to entrapped air bubbles [31]. This new method could be used for instance to assess the behavior of the connection before and after a mechanical stress mimicking the mastication strengths.

Gas permeability appears to be a new, simple, quantitative, reproducible and practical *in vitro *technique to assess dental implant-abutment leakage.

## Competing interests

The authors declare that they have no competing interests.

## Authors' contributions

All authors contributed to the work and the manuscript writing.
